# Malignant otitis externa presenting cerebral infarction from pseudoaneurysm: A case report and a review of the literature

**DOI:** 10.1002/ccr3.5276

**Published:** 2022-02-14

**Authors:** Yasutoshi Koshiba, Ryoukichi Ikeda, Jun Suzuki, Yohei Honkura, Yukino Funayama, Kensuke Ikeda, Hitoshi Warita, Masashi Aoki, Tetsuaki Kawase, Yukio Katori

**Affiliations:** ^1^ Department of Otolaryngology, Head and Neck Surgery Tohoku University School of Medicine Sendai Japan; ^2^ Department of Neurology Tohoku University School of Medicine Sendai Japan

**Keywords:** cerebral infarction, internal carotid artery, malignant otitis externa, pseudoaneurysm, skull base osteomyelitis

## Abstract

Chronic renal failure and diabetes mellitus could also be risk factors of pseudoaneurysm of the internal carotid artery (ICA) due to malignant otitis externa (MOE). Although pseudoaneurysm of the ICA is a rarely encountered disease, it should always be taken into consideration when treating patients of MOE.

## Graphical abstract

Although pseudoaneurysm of the internal carotid artery is a rarely encountered disease, it should always be taken into consideration when treating patients of malignant otitis externa.

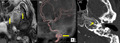

## 1 INTRODUCTION

A pseudoaneurysm of the internal carotid artery (ICA) is an uncommon complication of head and neck infections.^1^ They result from septic emboli, trauma, tumor invasion, fibromuscular, or iatrogenic causes. However, most cases are caused by the direct spread of local infections.^2^ There have been only five reports of petrous ICA pseudoaneurysm due to malignant otitis externa (MOE).^3^, ^4^, ^5^, ^6^, ^7^ Although rare, pseudoaneurysms of the ICA require prompt diagnosis and treatment to prevent hemorrhage and death.^4^ We experienced a rare case of MOE, leading to a pseudoaneurysm and cerebral infarction. Moreover, we reviewed the literature on petrous ICA pseudoaneurysm due to the otogenic infection.

## 2 CASE REPORT

**FIGURE 1:**
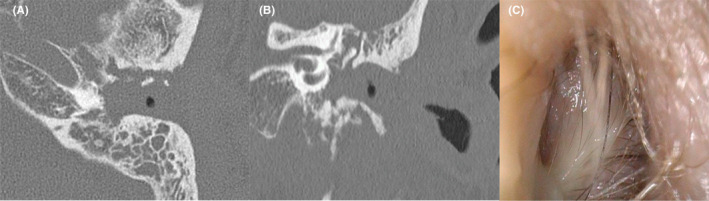
Computed tomography of the left temporal bone (A: Axial, B: Coronal). (C) Endoscopic findings of the external auditory canal

**FIGURE 2:**
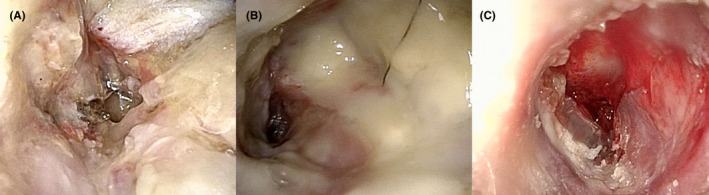
Endoscopic findings of the left ear. (A) Two weeks after operation. (B) Two weeks after discharge. (C) After readmission and treatment

A 78‐year‐old male patient with diabetes mellitus (DM) and chronic renal failure presented to the otolaryngology department at another hospital with left otalgia and otorrhea. On otoscopic examination using endoscopy, the anterior left external auditory canal (EAC) was swollen. He was suspected of Ramsay Hunt syndrome and treated with valtrex, cefditoren pivoxil, and ear drops of ofloxacin; however, his symptoms did not improve. Computed tomography (CT) imaging showed bone erosion of the EAC (Figure 1A), and EAC polyp's biopsy revealed granulation tissue (Figure 1C). Initial otorrhea culture showed *Pseudomonas aeruginosa* sensitive to levofloxacin (minimum inhibitory concentration (MIC) < 0.05) and tazobactam/piperacillin (MIC = 4). He was diagnosed with MOE. He was admitted to our hospital and started antibiotic treatment with tazobactam/piperacillin. The patient complained of headache, and a left facial nerve palsy (House Brackman Grade V) appeared. After two weeks of hospitalization, a canal wall down (CWD) mastoidectomy was performed. A postauricular incision was made, and a serous fluid reservoir was observed under the periosteum. Additionally, the subcutaneous tissue had thickened. The mastoid antrum and epitympanum were filled with granulation. The granulation around the facial nerve was not removed because nerve integrity monitor (NIM) showed a response at 0.4 mA. The ossicles were not manipulated, and the meatoplasty was performed. The patient was discharged with a tendency to improve in earache (Figure 2A). Levofloxacin was prescribed after discharge.

**FIGURE 3:**
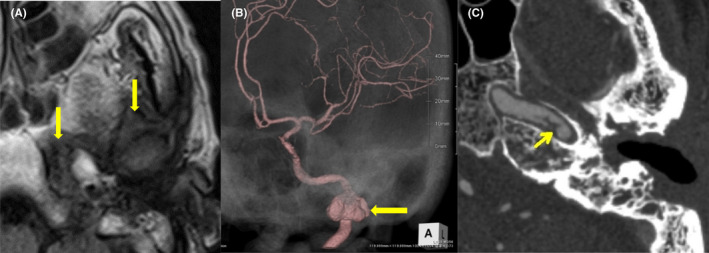
(A) Magnetic resonance imaging (T2‐weighted image) showed low intensity (yellow arrow) extending from the left external auditory canal to the pyramidal bone, temporomandibular joint fossa, and masticator space. (B) Magnetic resonance angiography revealed irregular dilatation of the left internal carotid artery. (C) Computed tomography angiography indicated an irregular mass in the left internal carotid artery

Two weeks after discharge, the patient became aware of weakness in the right upper and lower limbs during hemodialysis. Head magnetic resonance imaging (MRI) showed multiple cerebral infarctions in the left cerebral hemisphere. At the time of admission to our emergency department, the level of consciousness was Glasgow Coma Scale of 12 (eye‐opening: 4, best verbal response: 2, and best motor response: 6) and National Institutes of Health Stroke Scale score of 23. He also had aphasia, left concomitant deviation, right hemiplegia, and sensory disturbance. The MRI diffusion‐weighted image showed an acute embolic stroke in the left internal carotid artery region. There was an abnormal signal extending from the left EAC to the pyramidal bone, temporomandibular joint fossa, and masticator space. These findings suggested skull base osteomyelitis (Figure 3A). Magnetic resonance angiography (MRA) indicated irregular dilatation of the left internal carotid artery (Figure 3B). Additionally, arteriogenic embolism from the same area was suspected. CT angiography showed an irregular mass in the left internal carotid artery, which was considered pseudoaneurysm, and the petrous portion also shows wall irregularities (Figure 3C).

The tissue plasminogen activator (tPA) and thrombus retrieval were not indicated, and the patient was treated conservatively because it was more than 8 h after the stroke onset.

Treatment was started with vancomycin as MRSA had been detected in the most recent otolith culture (Figure 2B). The patient's treatment was then changed to cefepime and ceftazidime because three sets of blood cultures showed gram‐negative rods while *Pseudomonas aeruginosa* was identified. The fever quickly broke, and the inflammatory response decreased.

In parallel with the antibiotic treatment, the patient was treated with ear lavage, which settled the otorrhea and reduced the inflammatory reaction (Figure 2C); however, it did not improve consciousness and the paralysis of the upper and lower limbs. The patient was transferred to a convalescent hospital (Table 1).

**TABLE 1:**
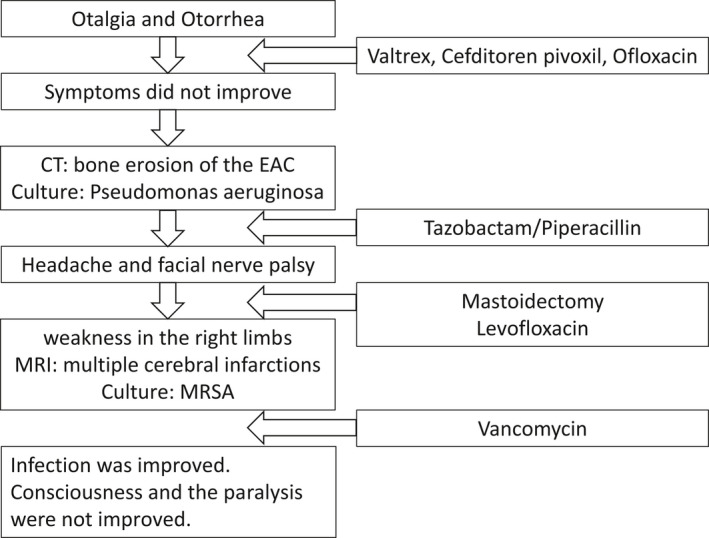
Medical flowchart

## 3 DISCUSSION

We have experienced a pseudoaneurysm of the ICA that developed from inflammation caused by MOE. MOE is an aggressive and rarely life‐threatening infection of the soft tissues of the external ear and surrounding structures. There have been six reported cases of pseudoaneurysm of the ICA due to MOE, including our case, and are summarized in Table 2.

**TABLE 2:** Summary of pseudoaneurysm of the internal carotid artery due to malignant otitis externa

Author	Kadkhodayan et al^3^	Baker et al^4^	Németh et al^5^	Chaudhary et al^6^	Lau et al^7^	Our case
Year	2012	2014	2017	2019	2019	
Age	50	81	68	66	59	78
Sex	F	M	F	M	M	M
Complication						
DM	−	+	+	+	+	+
Renal failure	+	−	−	−	+	+
Facial paralysis	−	−	+	−	+	+
Onset	Acute	Chronic	Acute	Chronic	Chronic	Acute
Hearing loss	−	+	−		+	−
Aural fullness	−	−	−	−	−	−
Tinnitus	−	+	−	−	+	−
Otalgia	−	+	+	+	−	+
Otorrhea	−	+	+	+	+	+
Other symptoms	Ear bleeding	Trismus, IX, X	Ear bleeding	Mastoiditis, parotid abscess	V, XII	−
ENT operation	−	−	Mastoidectomy	efss	−	Mastoidectomy
Treatment	Pipeline embolization	Coil embolization	Surpass flow diverter	Endovascular catheter embolization	Coil embolization	−
Antibiotics	+	Cefepime	+	Piperacillin/tazobactam, ciprofloxacin, amphotericin B		Tazobactam/Piperacillin, Vancomycin, ceftazidime
Bacteria	−	*P. aeruginosa*	+	*Candida glabrata*	*P. aeruginosa*	*P. aeruginosa*
Location of pseudoaneurysm	Petrous Internal carotid artery	Internal carotid artery	Surpass	Branches of the maxillary artery	Internal carotid artery	Internal carotid artery

The most common risk factor for developing MOE is DM, with being estimated that 90%–100% of patients with MOE have DM. Another risk factor is immunosuppression, such as patients suffering from human immunodeficiency virus (HIV), transplant patients, or patients with advanced cancer.^8^ In our summary of past cases, chronic renal failure requiring hemodialysis was observed in three cases (50%). Chronic renal failure patients have a high incidence of EAC cholesteatoma.^8^ Chronic renal failure could also be a risk factor for pseudoaneurysm of the ICA due to MOE.

The most common microbiological agent of MOE is *Pseudomonas aeruginosa*, followed by other pathogens such as *Proteus mirabilis*, *Aspergillus fumigatus*, *Proteus* spp., *Klebsiella* spp., and *Staphylococci* that have also been reported.^9^ Three cases were *Pseudomonas aeruginosa*, and one case was *Candida glabrata* in our review.

Cranial nerve involvement is commonly seen as part of aggressive MOE. Facial nerve palsy is most frequently encountered since it is near the external auditory canal. Facial nerve palsy increases mortality by 50%.^10^ Peled et al. reported that in 83 affected ears, five patients (6.0%) were presented with facial palsy. Our review demonstrated that three patients (50%) had facial paralysis, and two patients had other cranial nerve involvement (IX, X, V, and XII). These results suggest that in cases of cranial nerve palsy in MOE, vascular imaging is crucial to exclude pseudoaneurysms.

Conservative treatment is usually recommended for MOE. However, surgery is considered in aggressive or advanced disease, facial nerve paralysis, deep tissue sterile culture, and refractory MOE. In this patient, we performed surgery because of no clinical improvement after two months of conventional treatment and facial nerve paralysis. Surgery can reduce local infective load, remove necrotic tissue, and allow new tissue growth, which increases local vascularity, allowing systemic antibiotics to reach the critical area.^11^

Peled et al. suggested that in selected cases where minimal bone erosion is seen on CT, soft tissue debridement followed by post‐operative antibiotic treatment can be sufficient to achieve disease control. Extensive bone erosion requires CWU mastoidectomy and should be converted to CWD mastoidectomy in severe bone erosion of the posterior canal wall and when better exposure of the middle ear is required.^12^ CWD mastoidectomy was conducted; however, facial nerve decompression was not performed because the horizontal portion of the facial nerve was surrounded by granulation in our case. The mechanisms by which the infection could reach the arterial lumen are possibly hematogenous seeding or invasion of the adventitia of the artery from surrounding infection.^13^ The interventions include surgical resection and primary anastomosis, balloon occlusion, endovascular embolization/coiling, or stent placement.^13^ An endovascular approach is frequently adopted in the treatment of ICA pseudoaneurysm because expertise and accessibility in open surgery are limited. In this case, the appropriate timeframe for administration of acute treatment had passed, resulting in conservative treatment being chosen.

## 4 CONCLUSION

MOE is a severe condition that can lead to serious consequences. Although pseudoaneurysm of the ICA is a rarely encountered disease, it should always be taken into consideration when treating patients of MOE since it is a life‐threatening complication.

## CONFLICT OF INTEREST

The authors declare no financial relationships or conflict of interest.

## AUTHOR CONTRIBUTIONS

YK and RI involved in study concepts, data collection, data interpretation, and manuscript writing. JS, YH, YF, KI, and HW involved in data interpretation and manuscript revision. MA, TK, and YK edited the draft and reshaped it into this manuscript. All authors approved the final version of the manuscript and agreed to be accountable for all aspect of the work in ensuring that question related to the accuracy or integrity of any part of the work is appropriately investigated and resolved.

## ETHICAL APPROVAL

This study was approved by the Tohoku University Hospital Institutional Review Board (IRB protocol number: 20103).

## CONSENT

Written informed consent was obtained from the patient for publication of this case report and accompanying images.

## Data Availability

The data that support the findings of this study are available from the corresponding author upon reasonable request.
